# Genetic alterations in mature B- and T-cell lymphomas – a practical guide to WHO-HAEM5

**DOI:** 10.1515/medgen-2024-2005

**Published:** 2024-03-06

**Authors:** Cristina López, Anja Fischer, Andreas Rosenwald, Reiner Siebert, German Ott, Katrin S. Kurz

**Affiliations:** Universität Ulm und Universitätsklinikum Ulm Institut für Humangenetik Ulm Germany; Institut d’Investigacions Biomèdiques August Pi i Sunyer (IDIBAPS) Molecular Pathology Laboratory Barcelona Spain; Universität Würzburg Institut für Pathologie Würzburg Germany; Universität Ulm und Universitätsklinikum Ulm Institut für Humangenetik Ulm Germany; Robert-Bosch-Krankenhaus Abteilung für Klinische Pathologie Stuttgart Germany; Robert-Bosch-Krankenhaus Abteilung für Klinische Pathologie Stuttgart Germany

**Keywords:** B- and T-cell lymphomas, chromosomal rearrangements, somatic variants, copy number alterations, prognostic factors

## Abstract

The identification of recurrent genomic alterations in tumour cells has a significant role in the classification of mature B- and T-cell lymphomas. Following the development of new technologies, such as next generation sequencing and the improvement of classical technologies such as conventional and molecular cytogenetics, a huge catalogue of genomic alterations in lymphoid neoplasms has been established. These alterations are relevant to refine the taxonomy of the classification of lymphomas, to scrutinize the differential diagnosis within different lymphoma entities and to help assessing the prognosis and clinical management of the patients. Consequently, here we describe the key genetic alterations relevant in mature B- and T-cell lymphomas.

Owing to recent advances in the application of next generation sequencing (NGS) strategies, specific molecular alterations – although in part overlapping – have been associated with distinct clinico-pathologic entities of mature B-cell and T/NK-cell lymphoproliferations. These new insights have not only broadened our knowledge on ways of transformation but can also be of help in equivocal cases which may be difficult to classify solely on morphologic and immunophenotypic grounds. The updated World Health Organization classification of haematolymphoid neoplasms (WHO-HAEM5) used these molecular data to improve the classification of mature lymphoid neoplasms [1].

## Mature B-cell lymphomas

**Figure 1:**
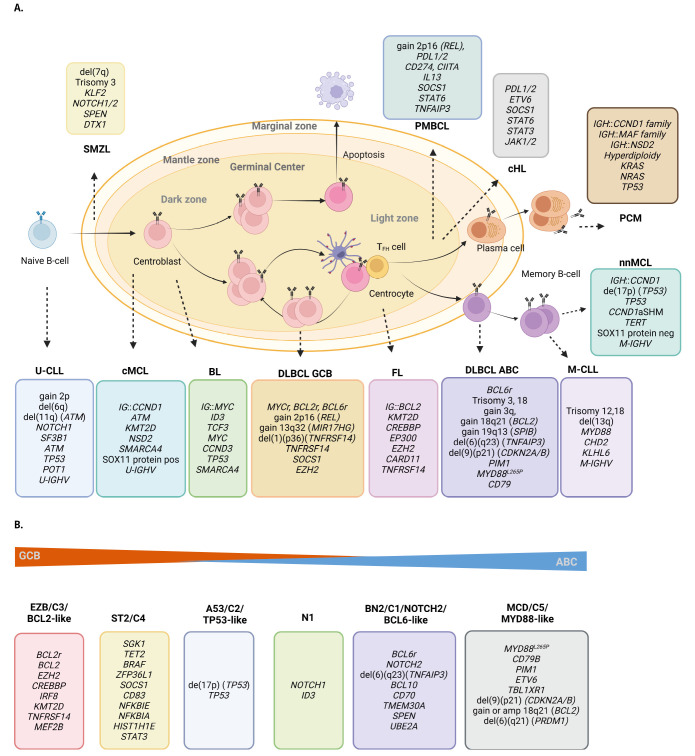
**Mature B-cell neoplasms, cell of origin and main genetic aberrations.** **A.** B-cell maturations process from the naïve B-cell to memory and plasma B-cell, and their derived B-cell lymphomas. The hallmark translocation and most frequently altered gene or genomic region are included in the box belonging to each entity. Of note, r, denotes rearrangement, genes in brackets are the candidate gene altered by the copy number change; and region/gene indicates that the gene is altered via copy number and/or somatic variants. U, unmutated; M, mutated, CLL, chronic lymphocytic leukaemia; cMCL, conventional mantle cell lymphoma; BL, Burkitt lymphoma; DLBCL-GCB, diffuse large B-cell lymphoma germinal centre subtype; FL, follicular lymphoma; DLBCL-ABC, diffuse large B-cell lymphoma activated subtype; PCM, plasma cell neoplasm; cHL, classical Hodgkin lymphoma; PMBCL, primary mediastinal large B-cell lymphoma; SMZL, splenic marginal zone lymphoma; neg, negative; pos, positive **B.** The genetic subtypes of DLBCL and the hallmark genetic aberrations of each subtype. Created with BioRender.com

The mature B-cell lymphomas are categorized in 12 families (**Figure 1A**).

**Preneoplastic and neoplastic small lymphocytic proliferations** comprise three subytpes of **monoclonal B-cell lymphocytosis (MBL).** Cases with low-count MBL frequently harbour 13q deletions. In contrast, high count MBL display the same incidence of chromosomal alterations typically encountered in CLL such as deletions of 11q22, 13q14, 17p13, and trisomy 12.

**Chronic lymphocytic leukaemia/small lymphocytic lymphoma (CLL/SLL),** is described in detail in a separate manuscript of this special issue.

## Splenic B-cell lymphomas/leukaemias

The family/class of splenic B-cell lymphomas/leukaemias encompasses Hairy cell leukaemia, Splenic marginal zone lymphoma, Splenic diffuse red pulp small B-cell lymphoma and Splenic B-cell lymphoma with prominent nucleoli. Hairy cell leukaemia (HCL) is characterized by the *BRAF^V600E^* mutation in virtually all cases. Loss of chromosome 7q, affecting *BRAF* has also been identified, as have somatic variants in *CDKN1B*, *KMT2C*, and *CCND3* [2]. The most frequent chromosomal alteration in **splenic marginal zone lymphoma** (SMZL) is 7q deletion, with a minimal deleted region involving 7q31-q32. Trisomy 3 or 3q gains are also detected in a mutually exclusive pattern with 7q deletions. No hallmark rearrangements are observed in SMZL, but some recurrent translocations such as *IG*::*CDK6* have been rarely identified. The most frequently altered genes are *KLF2* and genes related to the NOTCH pathway such as *NOTCH2*, *SPEN*, *NOTCH1* and *DTX1* [3]. On the other hand, in **splenic diffuse red pulp small B-cell lymphoma,** somatic mutations in *BCOR*, *CCND3*, *MAP2K1*, and genes of the NOTCH pathway are frequently identified, but the prevalence of 7q deletion is lower as compared to SMZL [4]. **Splenic B-cell lymphoma/leukaemia with prominent nucleoli (SBLPN)** of WHO-HAEM5 comprises the former HCL variant (HCLv) of WHO-HAEM4R, cases of CD5 negative B-prolymphocytic leukaemia (B-PLL), and a subset of SMZL. SBLPN lacks the *BRAF^V600E,^* but some cases harbour *MAP2K1* mutations [5].

**Lymphoplasmacytic lymphoma (LPL)** is characterized by *MYD88* (most of them *MYD88^L265P^* variant) and *CXCR4* somatic mutations in around 96% and 35% of patients with IgM LPL/Waldenström Macroglobulinemia (WM) [6]. LPL lacking *MYD88* mutations are characterized by activating mutations in the NF-κB pathway. *CXCR4* mutations are frequently subclonal events. *TP53* mutations are rare in LPL/WM but are associated with inferior clinical behaviour. Recurrent deletions in 6q encompassing *PLEKHG1*, *HIVEP2*, *ARID1B* and *BCLAF1* have been described in LPL/WG [6].

## Marginal zone lymphoma

**Extranodal marginal zone lymphoma of mucosa associated lymphoid tissue (EMZL)** shows different frequencies of genomic aberrations in the primary sites of origin. The translocations t(11;18)(q21;q21)/*BIRC3*::*MALT1,* t(14;18)(q32;q21)/*IGH*::*MALT1* and* t(1;14)(p22;q32)IGH::BCL10* are detected in a mutually exclusive pattern, and all of them activate the NF-κB pathway. Gains involving chromosomal regions 3/3q, 18/18q and 6p, and loss of 6q23 (*TNFAIP3*) are frequent. The landscape of somatic mutations is also depending on the site of origin; e. g. *TBL1XR1* mutations are more frequently detected in ocular adnexal lymphomas. Somatic variants in *PTPRD* and *BRAF* genes are highly prevalent in **nodal marginal zone lymphoma** (NMZL). Recurrent somatic variants in *KMT2D*, *NOTCH2*, *KLF2*, *TNFRSF14*, *TET2* and *CREBBP* have been observed. **Paediatric marginal zone lymphoma** is characterized by recurrent somatic variants in *MAP2K1*, *TNFRSF14*, and *IRF8* [7]*.*

## Follicular lymphoma

**Figure 2:**
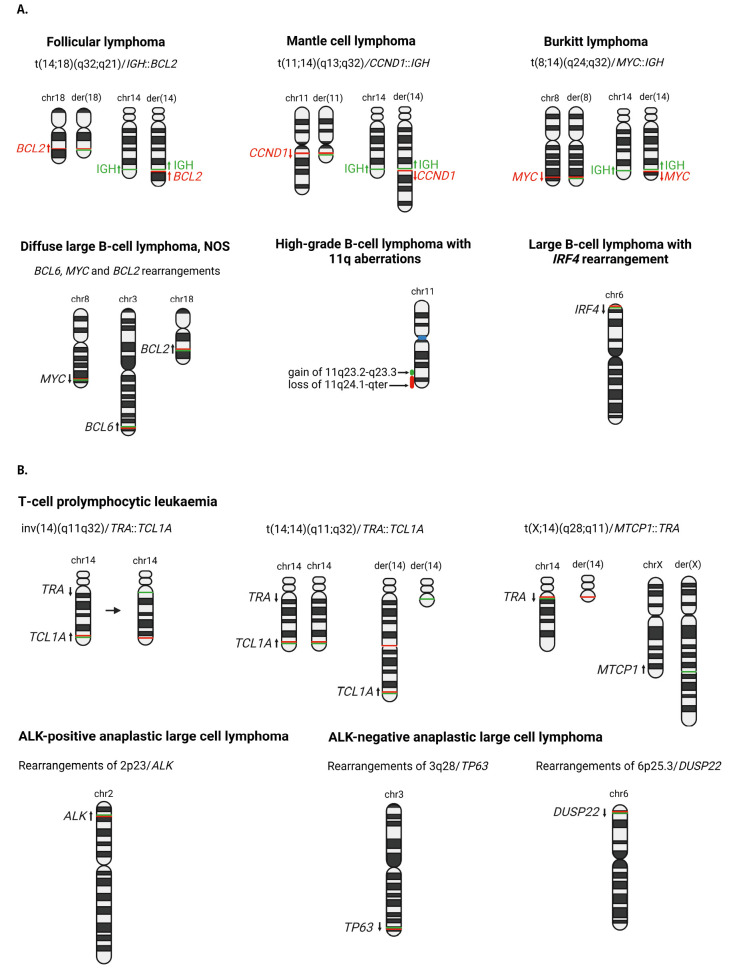
** Chromosomal alterations in mature B- and T-cell neoplasms. A**. Chromosomal alterations observed in specific entities of mature B-cell lymphomas. The rearranged genes are labelled according to the colour design of FISH dual colour dual fusion probes for the translocations t(14;18), t(11;14) and t(8;14), FISH break-apart probes for the loci *MYC*, *BCL2*, *BCL6* and* IRF4*, and locus specific FISH probes for the 11q aberrations. **B.** Chromosomal rearrangements detected in entities of T-cell neoplasms. The rearranged genes are labelled based on the colour design of FISH break-apart probes for the loci *TCL1A*, *TRA*, *ALK*, *TP63* and *DUSP22*. For the inv(14), t(14;14) and t(X;14), the genomic rearrangements and the derivative chromosomes generated from the chromosomal rearrangements are represented. Created with BioRender.com.

The genetic hallmark of classic follicular lymphoma (cFL) is the translocation t(14;18)(q32;q21)/*IGH*::*BCL2*, or its variants, leading to BCL2 overexpression (**Figure 2A**). Frequent losses of 1p, 6q, 10q/*PTEN*, 13q, and gains of 1q, 2p, 8q, 12q, and 18q, and trisomies of chromosomes 7, 18 and X have been identified in cFL. The most frequently altered genes in FL are involved in epigenetic and transcriptional regulation (*KMT2D*, *CREBBP*, *EP300*, and *EZH2*), JAK-STAT and NOTCH signalling (*SOCS1*, *NOTCH1*, *NOTCH2*), immune evasion (*TNFRSF14*), the BCR/NF-κB pathway (*CARD11*, *TNFAIP3*, *CD79A*, *CD79B*, and *MYD88*), and proliferation/apoptosis (*BCL2* and *TP53*) [8]. Of note a subtype of FL with diffuse growth pattern and CD23 expression lacks *BCL2* translocation, harbours deletions in 1p36/*TNRSF14* and has frequent somatic mutations in *STAT6* and *TNRFRSF14* [9]. In contrast, **paediatric-type follicular lymphoma** shows recurrent somatic mutations in the MAPK pathway and the *IRF8* gene and absence of *BCL2*, *BCL6*, *MYC* and *IRF4* rearrangements[8]. **Duodenal-type follicular lymphoma** harbours *BCL2* translocation and displays somatic mutations in *TNFRSF14*, *EZH2*, *KMT2D* and *CREBBP* [10].

## Mantle cell lymphoma (MCL)

Mantle cell lymphoma (MCL) is characterized by the translocation t(11;14)(q13;q32)/*CCND1*::*IGH*, leading to constitutive overexpression of CCND1, a cell cycle regulator (**Figure 2A**). A small fraction of MCL lacks *CCND1* rearrangements but harbours alternative *CCND2* or *CCND3* rearrangements, frequently involving the IG light chain genes. MCL comprises two molecular subtypes, conventional MCL (cMCL) and leukaemic non-nodal MCL (nnMCL). cMCL are characterized by unmutated IGHV, overexpression of SOX11, and high genomic complexity. In contrast, nnMCL display mutated IGHV, lack expression of SOX11 and have a lower number of genetic alterations. The genomic profile of MCL is characterized by losses in 1p22-p13, 6q, 9p21 (*CDKN2A/B*), 8p, 9q22-q31, 10p15-p13, 13q14 (*RB1*), 13q33-q34, 19q, and gains in 3q25-q29, 7p, 8q24 (*MYC*), 10p12 (*BMI*), and 13q31 (*MIR17HG*)[11]. The mutational landscape of MCL is defined by alterations in genes involved in cell cycle control (*CCND1*, *CDKN2A*/*B*, *CDK4*, and *RB1*), DNA damage response (*TP53*, *ATM*, and *MYC*), epigenetic modulators (*KMT2D*, *SMARCA4*, and *NDS2*), NOTCH pathway (*NOTCH1* and *NOTCH2*), and NF-κB signalling (*BIRC3*, *NFKBIE*, and TNFAIP3)[11]. Importantly, differences in the distribution of specific alterations have been identified between cMCL and nnMCL, e. g. loss of 17p/*TP53* is enriched in nnMCL, and somatic mutations in *ATM*, *KMT2D*, *NSD2* have been identified exclusively in cMCL [11].

## Transformations of indolent B-cell lymphomas

Although the genomic profile of transformed indolent B-cell lymphomas is partially defined by the specific genomic alterations of the preceding indolent lymphoma, there are key genes that are commonly altered and associated with the transformation. These genes are mainly affecting the proliferation and survival pathways (*MYC* rearrangement or amplification, *CDKN2A/B* deletions*, TP53* deletions/mutations), and NF-κB (*TNFAIP3* deletions/mutations).

## Large B-cell lymphomas

**Diffuse large B-cell lymphoma (DLBCL), NOS,** comprises two molecular subtypes based on gene expression profiling, germinal center B-cell like (GCB) and activated B-cell like (ABC), reflecting their different cell of origin. These subtypes are associated with different genomic alterations and clinical behaviour. *IGH*, *BCL2*, *BCL6* and *MYC* rearrangements are detected in approximately 40%, 20%, 20–40% and 10% of the cases, respectively (**Figure 2A**) In the case of *MYC* translocations, deregulation can occur through juxtaposition to an enhancer element of an IG locus (*IG*::*MYC*) but also regulatory elements of other genes (non-*IG*::*MYC*). *BCL2* rearrangements mostly involve an IG locus, predominately IGH, as partner and is mainly caused by an aberrant VDJ rearrangement. In contrast, alterations of the *BCL6* locus are quite promiscuous and enhancer hijacking can involve a large number of partners. *MYC* and *BCL6* alterations are seen in both subtypes while *BCL2* rearrangements are frequently translocated in GCB subtype. The ABC subtype is characterized by gains of 3/3q, 18/18q21 (*BCL2*), 19q13 (*SPIB*), and losses of 6q23 (*TNFAIP3)* and 9p21 (*CDKN2A/2B*), and somatic variants affecting* PIM1*, *MYD88^L265P^*, and *CD79B.* The GCB subtype is defined by gains of 2p16/*REL* and 13q32/*MIR17HG*, trisomies of chromosomes 7 and 12, deletions of 1p36/*TNFRSF14* and 10q23/*PTEN,* and somatic variants in* TNFRSF14*, *SOCS1*, *EZH2*[12]*.* This molecular subclassification, however, only incompletely captures the molecular heterogeneity of DLBCL. Consequently, more recent large scale molecular studies described 5 to 6 genetic subgroups: i) BN2/C1/BCL6-like; ii) EZB/C3/BCL2-like; iii) MCD/C5/MYD88-like; iv) C2/A53/TP53-like; v) C4/ST2; vi) N1 (**Figure 1B**) [12, 13].

**Diffuse large B-cell lymphoma/high grade B-cell lymphoma with** ***MYC* and** ***BCL2* rearrangements (DLBCL/HGBL-*MYC*/*BCL2*)**. Concomitant *MYC* and *BCL2* translocations are the genetic hallmark of this entity. An IG locus is the *MYC* partner in 50% of the cases, the most common being t(8;14)(q24;q32)/*IGH*::*MYC.* The mutational profile of these lymphomas is characterized by somatic variants in genes commonly altered both in FL and transformed lymphomas or Burkitt lymphoma (BL) such as *CREBBP*, *KMT2D*, *EZH2*, *SI*, *TNFRSF14, BCL2, MYC, CCND3* and *TP53* [14, 15].

**Large B-cell lymphoma with** ***IRF4* rearrangement (LBCL-*IRF4*)** is defined by an *IRF4* rearrangement, the most frequent being t(6;14)(p25;q32)/*IRF4*::*IGH* (**Figure 2A**)*.* Less frequently, *IRF4* is rearranged with one of the IG light chain genes*.* In some cases, a *BCL6* rearrangement can be detected in addition, whereas *MYC* or *BCL2* translocations are absent. Mutations in *IRF4* and alterations in NF-κB related genes (*CARD11*, *CD79B,* and *MYD88*) have been described [16].

**High-grade B-cell lymphoma with 11q aberrations** is characterized by a gain of 11q23.2-q23.3 and a loss (or copy number neutral loss of heterozygosity in occasional cases) of 11q24.1-qter (**Figure 2A**). The cases display morphological and immunophenotypic features similar to BL, but the genetic profile is distinct. *MYC* translocations are by definition absent and no rearrangements of *BCL2* nor *BCL6* are identified. Somatic variants in *BTG2*, *DDX3X*, *ETS1*, *EP300*,* NFRKB* and *GNA13* have been detected [17, 18].

**Primary large B-cell lymphoma of immune-privileged sites** represents a new umbrella entity in WHO-HAEM5. It includes primary DLBCL of the central nervous system (CNS), the vitreoretinal compartment, and the testes of immunocompetent patients. Tumours in this umbrella category are characterized by a similar high prevalence of somatic variants in *PIM1*, *MYD88^L265P^*, and *CD79B.* Furthermore, genomic alterations in genes involved in immune evasion, such as rearrangements in the *PD-L1*/*PD-L2* loci (9p24), and loss of *HLA* loci (6p21), and DNA damage, *CDKN2A/B*(9p21) are key players [19]. The genomic landscape of this entity corresponds to molecular subgroups MCD/C5 [12]. Notwithstanding this, various mutational processes contribute to the pathogenesis of primary DLBCL of the CNS [20, 21].

**Primary mediastinal large B-cell lymphoma (PMBCL)** is characterized by activation of JAK-STAT and NF-κB signalling pathways. JAK-STAT is activated via activation of IL13, loss of function mutations in *SOCS1* and *PTPN1*, and gain of function mutations in *STAT6* and *IL4R*. The NF-κB pathway is altered via gains in 2p16.1/REL and inactivating mutations of negative regulators such as *TNFAIP3* or *NFKBIE* [22]. In addition, genetic alterations involve genes related to immune evasion such as *PD-L1*/*PD-L2*, *CD58*, *B2M*, *CD274* and *CIITA.* The genetic profile is in proximity to classic Hodgkin lymphoma.

**Mediastinal gray zone lymphoma (MGZL)** displays characteristics intermediate between PMBCL and classic Hodgkin lymphoma (CHL). MGZL is characterized by somatic variants in *SOCS1*, *B2M*, *TNFAIP3*, *GNA13*, *LRRN3* and *NFKBIA*[23]. This mutational profile is specific of the thymic niche and different from morphologically similar tumours arising in extra-mediastinal regions. A *BCL2* rearrangement is present in only 6% of the cases and *BCL6* translocations are virtually absent.

**High-grade B-cell lymphoma, NOS (HGBL, NOS)** is a biologically heterogenous entity. *MYC* translocations are identified in 50% of the cases, whereas *BCL2* (without *MYC* translocation by definition) and *BCL6* rearrangements are less prevalent. No specific mutational profile has been identified in these patients, although somatic variants in genes altered in BL such as *ID3*, *CCND3* and *MYC* or genes altered in DLBCL, NOS including *CREBBP* and *BCL2* have been reported [24].

## Burkitt lymphoma

A *MYC* rearrangement is the hallmark aberration in BL leading to MYC overexpression. The translocation t(8;14)(q24;q32)/*IGH*::*MYC* is present in approximately 80% of the patients (**Figure 2A**), and, less frequently, *MYC* is rearranged with the IG light chain loci [25]*.* Deregulation of the *ID3*-*TCF3* axis is highly specific of BL. *ID3* and *TCF3* alterations are detected in 10–20% and 50–60% of the patients, respectively. Additional somatic variants detected in BL affect *MYC,* *CCND3*, *TP53*, *SMARCA4,* and *GNA13* genes*.* Overall, four pathways are altered in BL*,* BCR signalling, proliferation and survival, sphingosine-1-phosphate signalling, and SWI-SNF chromatin remodelling [25]. Recent molecular studies support the idea to subclassify BL based on EBV status rather than the traditional epidemiological variants. EBV-BL is characterized by a high frequency of *ID3*, *TCF3* and *CCND3* somatic alterations whereas EBV+ BL is defined by a higher number of somatic variants and increased PI3K activity [26]. BL may occur in paediatric and adult patients and a recent study reported that genomic alterations such as *ID3*, *DDX3X*, *ARID1A*, and *SMARCA4* were more frequent in paediatric patients whereas *BCL2* and *YY1AP1* have been reported to be particularly altered in adult patients [27].

## Hodgkin lymphoma (HL)

Hodgkin lymphoma (HL) is divided in two main forms,** classic Hodgkin lymphoma (cHL)** and **nodular lymphocyte predominant Hodgkin lymphoma (NLPHL)**. Both forms show dysregulation of the JAK-STAT pathway (*STAT6*, *STAT3*, *JAK1*/*2*, *SOCS1*), NF-κB (*TNFAIP3)*, and immune evasion pathways (*PDL1*/*2*, *B2M*, *ETV6*, and *CIITA)* [28]. Nevertheless, NLPHL shows also aberrations common in other B-cell lymphomas, like *BCL6* rearrangements.

**Plasma cell neoplasms and other diseases with paraprotein** are described in detail in another manuscript of this special issue.

## T-cell and NK-cell lymphomas

**Figure 3:**
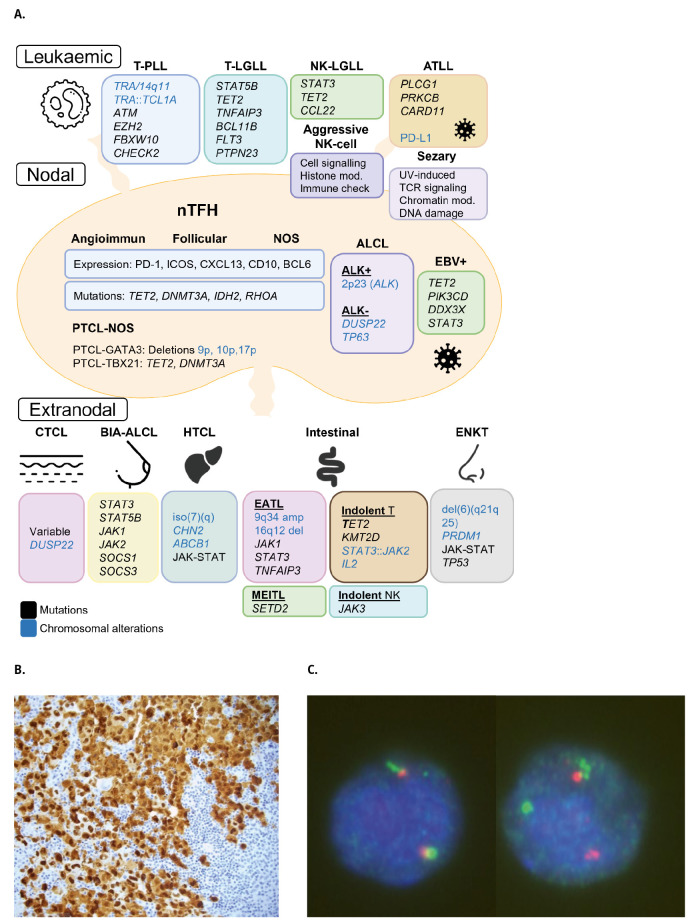
**Main genetic aberrations in T/NK-cell malignancies and** ***ALK* rearrangements in Anaplastic large cell lymphomas.** **A.** The mature subgroup of T/NK-cell malignancies is divided into leukaemic, nodal and extranodal. The most frequently altered genes or genomic regions are included in the box belonging to each entity. T-PLL, T-Prolymphocytic leukaemia; T-LGLL, T-large granular-lymphocytic leukaemia; NK-LGLL, NK-large granular lymphocytic leukaemia; ATLL, Adult T-cell leukaemia/lymphoma; nTFH, Nodal T-follicular helper; PTCL-NOS; peripheral T-cell lymphomas not otherwise specified; ALCL, Anaplastic large cell lymphoma; CTCL, Cutaneous T-cell lymphoma; BIA-ALCL, Breast implant-associated ALCL; HTCL, Hepatosplenic T-cell lymphoma; EATL, Enteropathy-associated T-cell lymphoma; MEITL, monomorphic epitheliotropic intestinal T-cell lymphoma; ENKT, extranodal NK/T-cell lymphoma. **B.** Staining of ALK using immunohistochemistry showed a large proportion of ALK positive cells. **C.** Fluorescence in situ hybridisation using an *ALK* break-apart probe. A cell without break is shown on the left and an ALK-positive cell on the right, showing one fusion signal (green & red signals) corresponding to the unarranged allele, and one green and one red signal indicating a break in *ALK* gene.

T-cell and NK-cell lymphoproliferations and lymphomas are organized in 8 families in WHO-HAEM5. **Figure 3A** provides an overview on genetic alterations in different entities of T-cell lymphomas in WHO-HAEM5.

## Mature T-cell and NK-cell leukaemias

Chromosomal rearrangements involving 14q11 in **T-Prolymphocytic leukaemia** (T-PLL) deregulate TCL1A due to a *TRA*::*TCL1A* rearrangement (**Figure 2B**). In a small subsets of T-PLL *TCL1A* is substituted by *MTCP1* on chromosome X; in addition, T-PLL is often characterized by mutations and/or deletions of *ATM, EZH2, FBXW10* and *CHEK2* genes [29]. **T-large granular-lymphocytic leukaemia** (T-LGLL) is characterized by recurrent *STAT5B* mutations and other recurrently altered genes such as *TET2, TNFAIP3, BCL11B, FLT3* und *PTPN23* [30]. **NK-large granular lymphocytic leukaemia**, in contrast, shows activating mutations of *STAT3, TET2* and *CCL22*. **Adult T-cell leukaemia/lymphoma (ATLL)**, next to its classical association with HTLV-1, shows a plethora of activating mutations such as *PLCG1, PRKCB* and *CARD11* among others and, notably, also structural variations of PD-L1 [31]. **Sezary syndrome** characteristically displays a high mutational burden inferred by UV-induced damage and mutations affecting TCR-signalling, chromatin-modifier, and DNA-damage-response pathways. Finally, **aggressive NK-cell leukaemia** is characterized by mutations in genes affecting cell signalling, histone modifying molecules and immune checkpoint molecules.

## Primary cutaneous T-cell lymphomas

The genetic constitution of **Cutaneous T-cell neoplasms** is highly variable, reflecting their highly variable clinical presentation and morphological features. More recent literature has identified rearrangements of the *DUSP22-IRF4* locus in a small subset of lymphomatoid papulosis cases, originally described as a characteristic of nodal ALK-negative anaplastic large cell lymphoma.

## Intestinal T-cell and NK-cell lymphoid proliferations and lymphomas

**Enteropathy-associated T-cell lymphoma (EATL)** and **monomorphic epitheliotropic intestinal T-cell lymphoma (MEITL)** represent distinct entities, the former being strongly associated with celiac disease. Their genetic features are similar, however not identical, with amplifications of 9q34 and deletions at 16q12 seen in a high proportion of EATL, as are activating mutations in *JAK1* and *STAT3*. Loss-of-function mutations in *TNFAIP3* result in NF-kB activation [32]. In MEITL, next to JAK-STAT deregulation, mutations of *SETD2*, a histone-lysin 36 methyltransferase obviously is crucial for disease initiation and propagation. **Indolent T-cell-lymphoma of the gastrointestinal tract (GIT)** harbours mutations in epigenetic modifying genes such as *TET2* and *KMT2D*, recurrent *STAT3*::*JAK2* fusions and structural alterations of the *IL2* gene; interestingly, these alterations are found in different frequencies in CD4-positive and CD8-positive (or double negative) subsets. The finding of recurrent mutations in *JAK3* and other genes has built evidence that **Indolent NK-cell lymphoproliferation (NKLPD) of the GIT** is a neoplastic disease, however, basing on its generally indolent clinical course, this disease has been named “lymphoproliferative disorder” and not “lymphoma”.

## Hepatosplenic T-cell lymphoma

This lymphoma shows a prototypic isochromosome 7q which forms the basis of the overexpression of *CHN2, ABCB1* and other genes. Several mutations in genes of the JAK-STAT pathway and/or epigenetic modifier genes have been described.

## Anaplastic large cell lymphoma

Anaplastic large cell lymphoma (ALCL) denotes a group of mature T-cell lymphomas that are similar in their cytomorphology and immunophenotype but clinically and genetically heterogeneous (**Figure 2B**). ALCL showing rearrangements in 2p23, the location of the *ALK* gene, constitute 50 % of tumours (**Figure 3B-C**). In the translocations, more than 20 partner genes are involved deregulating ALK expression, the most frequent one being *NPM1* involved in the t(2;5)(p23;q35) [33]. Constitutive activation of the tyrosine kinase ALK activates several signalling pathways including PI3K/AKT, mTOR, JAK-STAT3 and STAT5B. In contrast, **ALK-negative ALCL** feature rearrangements of the *DUSP22* and *TP63* genes in around 30% and 5% of cases, respectively. These oncogenic fusions involving tyrosine kinase genes other than *ALK* also lead to STAT3 activation; transcription factors such as IRF4 and MYC have been shown to be essential for the survival of ALCL cells [33]. **Breast implant-associated ALCL (BIA-ALCL)** occurs approximately 10 years after implant placement and generally runs an indolent clinical course, especially if the lymphoma cells are confined to the peri-implant space and/or occur as an effusion. Although the mutational landscape of BIA-ALCL is not fully elucidated, activating somatic mutations have been described in *STAT3, STAT5B, JAK1* and *JAK2* and loss of function mutations in *SOCS1* and *SOCS3* pointing to the importance of activation of the JAK/STAT pathway also in this disease.

## Nodal T-follicular helper (TFH) cell lymphoma

An important new concept is the creation of an umbrella entity of nodal T-follicular helper cell lymphomas (nTFH lymphomas) encompassing **nTFH-angioimmunoblastic-type**, **nTFH-follicular-type** and **nTFH-NOS** that have different morphological features but in common a derivation from nodal helper T-cells of the reactive germinal center and an expression of TFH-associated immunophenotypic markers such as PD-1, ICOS, CXCL13, CD10, BCL6 and others[34]. Of importance, the gene expression profiles of these three nTFH-types are remarkably similar, as is their mutational landscape featuring mutations in *TET2, DNMT3A*, *IDH2* and *RHOA*, albeit at differing frequencies. Of importance, *TET2* and *DNMT3A* mutations have been shown to arise in myeloid precursor cells and are classical components of the concept of clonal haematopoiesis, whereas *IDH2* and *RHOA* formation has been shown to occur in somatic, T-cell – committed – tumour cells, thus linking clonal haematopoiesis with T-cell lymphomagenesis.

## Peripheral T-cell lymphomas, NOS

The genetic landscape of **peripheral T-cell lymphomas (PTCL), NOS**, is heterogeneous. Gene expression profiling studies have identified two molecular subtypes, PTCL-GATA3 and PTCL-TBX21, and these subtypes do also differ in their genetic landscapes with 9p, 10p and 17p deletions mainly occurring in PTCL-GATA3 and PTCL-TBX21 harbouring frequently mutations in epigenetic regulators such as *TET2* and *DNMT3A*.

## EBV-positive NK/T-cell lymphomas

Among the classically **EBV-positive T-cell and NK-cell lymphomas**, extranodal NK/T-cell lymphoma is frequently arising – as the name implies – in extranodal sites such as the nasal cavity and the paranasal sinuses, but also in the skin, GIT, testis and other locations. It is characteristically associated with deletions in 6q21-25 harbouring several tumour suppressor genes such as *PRDM1* and others and features recurrent mutations in JAK-STAT pathway genes, epigenetic regulators, tumour suppressor genes (such as *TP53*) and RNA helicases. In contrast, EBV-positive nodal T-cell and NK-cell lymphoma is a nodal neoplasm mainly diagnosed in Asian patients and often shows mutations in *TET2, PIK3CD, DDX3X* and *STAT3*[35].

## EBV-positive T-and NK-cell lymphoid proliferations and lymphomas of childhood

The family of **EBV-positive T-cell- and NK-cell lymphoid proliferations and lymphomas of childhood** represents a group of uncommon disorders characterized by EBV-infection of T- and NK-cells and shows variable clinical features ranging from indolent and localized forms to systemic disease characterized by constitutional symptoms and high clinical aggressiveness. The genetic landscape of these diseases has been incompletely characterized. Somatic mutations have been identified in *DDX3X* (e. g. in severe mosquito bite allergy suggesting that this is a neoplastic disease) and in systemic chronic active EBV disease [36].

## Conclusions

Genetic data can be helpful or are occasionally even essential in the diagnosis of, and sometimes highly relevant in treatment decisions in haematological malignancies (**Table 1**). The identification of specific chromosomal rearrangements using gold standard techniques such as conventional cytogenetics and molecular cytogenetics (FISH) are essential to identify hallmark aberrations specific to some subtypes of mature lymphomas, such as t(8;14)/*IGH*::*MYC* translocation in BL. Besides, NGS technologies have been important to identify somatic variants crucial in a subset of entities and defining new target vulnerabilities. Taken together, the identification of genetic alterations in mature B- and T-cell lymphomas have impact in the diagnosis, risk stratification, and ultimately clinical management, of the patients.

**Table 1:** Clinical relevance of genetic alterations in mature B and T-cell neoplasms

**Entities**		**Genetic alterations**	**Clinical relevance**
**Mature B-cell lymphomas**			
**Splenic B-cell lymphomas and leukaemias**			
	**Hairy cell leukaemia (HCL)**	*BRAF*^V600E^ mutation	Appropriate for diagnosis
	**Splenic marginal zone lymphoma (SMZL)**	del(7q), +3, +18 *KLF2*, *NOTCH2*, *SPEN*, *NOTCH1*, *DTX1* mutations	Useful to support diagnosis
	**Splenic diffuse red pulp small B-cell lymphoma (SDRPL)**	*BCOR*, *CCND3*, *MAP2K1* mutations	Useful to support diagnosis
	**Splenic B-cell lymphoma/leukaemia with prominent nucleoli (SBLPN)**	*MAP2K1* mutation	Associated with SBLPN or HCL variant, useful to support diagnosis
**Lymphoplasmacytic lymphoma**			
	**Lymphoplasmacytic lymphoma (LPL)**	del(6q)	Useful to support diagnosis
*CXCR4* mutations		Associated with resistance to ibrutinib treatment
*MYD88* (mainly *MYD88^L265^*^P^) mutations		Appropriate for diagnosis
*TP53* mutations		Associated with inferior clinical behaviour
**Marginal zone lymphoma**			
	**Extranodal marginal zone lymphoma of mucosa associated lymphoid tissue (EMZL)**	*BIRC3*, *BCL10* rearrangements Gains 3/3q, 18/18q, 6p Loss of 6q23 (*TNFAIP3*) *TBL1XR1* mutations	Useful to support diagnosis
*MALT1 rearrangements*		Associated with lack of response to antibiotic treatment in Helicobacter pylori-positive gastric EMZL
	**Nodal marginal zone lymphoma (NMZL)**	+3, +8 *KLF2, NOTCH2, PTPRD*, *BRAF* mutations	Useful to support diagnosis
	**Paediatric marginal zone lymphoma**	*MAP2K1, TNFRSF14, IRF8* mutations	Useful to support diagnosis
**Follicular lymphoma**			
	**Classic Follicular lymphoma (cFL)**	*IG*::*BCL2* translocation	Appropriate for diagnosis and useful for differential diagnosis of *BCL2* rearrangements negative FL
*EZH2* mutations		Useful to predict response to EZH2 inhibition (e. g. Tasemetostat)
	**Paediatric-type follicular lymphoma**	No* BCL2* rearrangements	Appropriate for diagnosis and useful for differential diagnosis of *BCL2* rearrangements positive FL (cFL)
*MAP2K1, TNFRSF14, IRF8* mutations.		Useful to support diagnosis
	**Duodenal-type follicular lymphoma**	*IG*::*BCL2* translocation	Appropriate for diagnosis
*TNFRSF14, EZH2, KMT2D and CREBBP* mutations		Useful to support diagnosis
**Mantle cell lymphoma**			
	**Mantle cell lymphoma**	*IG*::*CCND1* translocation	Appropriate for diagnosis
*CCND2* and *CCND3* rearrangements		Appropriate for diagnosis in *CCND1* negative MCL tumors
Loss 17p/*TP53* mutations		Associated with inferior clinical behaviour
	**Non-nodal mantle cell lymphoma (nnMCL)**	Loss 17p/*TP53* mutations	Useful to support diagnosis, enriched in nnMCL subtype
	**Conventional mantle cell lymphoma (cMCL)**	*ATM*, *KMT2D*, *NSD2* mutations	Useful to support diagnosis, exclusively in cMCL subtype
**Large B-cell lymphomas**			
	**Diffuse large B-cell lymphoma, NOS (DLBCL-NOS)**	*MYC*, *BCL2*, *BCL6* rearrangements Cases with dual *MYC* and *BCL6* translocations as a genetic subtype of DLBCL, NOS	Appropriate for diagnosis and useful for differential diagnosis of DLBCL/HGBL-*MYC*/*BCL2*
	**DLBCL, NOS GCB subtype**	Gains 2p16/*REL*, 13q32/*MIR17HG* Losses 1p36/*TNFRSF14*, 10q23/*PTEN* *TNFRSF14*, *SOCS1*, *EZH2* mutations	Useful to support diagnosis, enriched in DLBCL GCB subtype
	**DLBCL, NOS ABC subtype**	Gains 3/3q, 18/18q21 (*BCL2*), 19q13/*SPIB* Losses 6q23/*TNFAIP3*, 9p21/*CDKN2A*/*B* *PIM1*, *MYD88*^L265P^, and *CD79B* mutations	Useful to support diagnosis, enriched in DLBCL ABC subtype
	**Diffuse large B-cell lymphoma/high grade B-cell lymphoma with** ***MYC* and** ***BCL2* rearrangements (DLBCL/HGBL-*MYC*/*BCL2*)**	By definition dual *MYC* and *BCL2*, rearrangements. *BCL6* can be translocated or not.	Appropriate for diagnosis and useful for differential diagnosis of DLBCL, NOS
	**Large B-cell lymphoma with** ***IRF4* rearrangement (LBCL-*IRF4*)**	*IRF4* rearrangement (*IRF4* mutations)	Rearrangements appropriate for diagnosis and mutations useful to support diagnosis in cases of *IRF4* cryptic translocation
No* MYC* and* BCL2* rearrangements		Appropriate for diagnosis and useful for differential diagnosis of DLBCL, NOS
	**High-grade B-cell lymphoma with 11q aberrations (HGBL-11q)**	Gain 11q23.2-q23.3 Loss or CN-LOH 11q24.1-qter	Appropriate for diagnosis and useful for differential diagnosis of BL with cryptic *MYC* translocation
No *BCL6*, *MYC*, *BCL2* rearrangements		Appropriate for diagnosis and useful for differential diagnosis of other mature B-cell lymphomas (e. g. BL, HGBL, NOS)
*BTG2*, *DDX3X*, *ETS1*, *EP300*, *NFRKB, GNA13* mutations		Useful to support diagnosis
	**EBV positive diffuse large B-cell lymphoma**	Mutations in *CCR6, CCR7, DAPK1, TNFRSF21, CSNK2B* and *YY1.* 6q deletions (*PRDM1* and *TNFAIP3*).	Appropriate for diagnosis and useful for differential diagnosis of other EBV-associated LBCL
	**Primary large B-cell lymphoma of immune-privileged sites**	Gain or rearrangement *PD-L1*/*PD-L2* Losses 6p21 (*HLA*), 9p21 (*CDKN2A/B*) *PIM1*, *MYD88*^L265P^, and *CD79B*	Useful to support diagnosis
	**Primary mediastinal large B-cell lymphoma (PMBCL)**	Gains or rearrangement *PDL1/PDL2*, *CD274* Losses or mutations *CD58*, *B2M*, *CIITA* *STAT6*, *IL4R*, *TNFAIP3*, *NFKBIE* mutations	Useful to support diagnosis
	**Mediastinal gray zone lymphoma (MGZL)**	*SOCS1*, *B2M*, *TNFAIP3*, *GNA13*, *LRRN3* and *NFKBIA* mutations	Useful to support diagnosis
No* BCL6* rearrangement		Useful to support diagnosis
	**High-grade B-cell lymphoma, NOS (HGBL, NOS)**	*MYC*, *BCL2*, *BCL6* rearrangements. By definition no dual *MYC* and *BCL2* translocations. Cases with dual *MYC* and *BCL6* translocations as a genetic subtype	Appropriate for diagnosis
**Burkitt lymphoma**			
**Burkitt lymphoma (BL)**		*IG*::*MYC* translocation	Appropriate for diagnosis and useful for differential diagnosis of HGBL with 11q aberration
No* BCL2, BCL6* rearrangements	Appropriate for diagnosis and useful for differential diagnosis of HGBL, NOS and HGBL/*MYC*/*BCL2*
*ID3* and* TCF3* mutations	Useful to support diagnosis and for BL with cryptic *MYC* translocation
**Hodgkin lymphoma**			
	**Classic Hodgkin lymphoma (cHL) and nodular lymphocyte predominant Hodgkin lymphoma (NLPHL)**	Gains or rearrangement *PD-L1/PD-L2*	Useful for treatment with PD1 inhibitor in relapse/refractory cHL
**Mature T-cell lymphoma**			
**Mature T-cell and NK-cell leukaemia**			
	**T-prolymphocytic leukaemia/ lymphoma: (T-PLL), NOS**	inv(14) or t(14;14) /*TRA*::*TCL1* t(X;14)/*MTCP1*::*TRA*	Appropriate for diagnosis
	**T-large granular lymphocytic leukaemia (T-LGLL)**	*STAT5B* mutations	Useful for diagnosis
	**NK-large granular lymphocytic leukaemia (NK-LGLL)**	*TET2* and *CCL22* mutations	Useful for diagnosis
*STAT3* mutations		Associated with neutropenia
	**Adult T-cell leukaemia/lymphoma**	*PLCG1* and *CARD11* mutations	Useful for diagnosis
*TP53* and* PRKBC* mutations		Associated with inferior clinical behaviour
**Intestinal T-cell and NK-cell lymphoid proliferations and lymphomas**			
	**Enteropathy-associated T-cell lymphoma (EATL)**	Amplification 9q34 Loss 16q12	Appropriate for diagnosis
*JAK1, STAT3, TNFAIP3* mutations		Useful for diagnosis
	**Monomorphic epitheliotropic intestinal T-cell lymphoma (MEITL)**	*SETD2* mutations	Useful for diagnosis
	**Indolent T-cell-lymphoma of the gastrointestinal tract (GIT)**	*STAT3*::*JAK2* *TET2, KMT2D* mutations	Useful for diagnosis
	**Indolent NK-cell lymphoproliferation (NKLPD) of the GIT**	*JAK3* mutations	Useful for diagnosis
**Hepatosplenic T-cell lymphoma**			
	**Hepatosplenic T-cell lymphoma (HSTCL)**	i(7q)	Useful for diagnosis
**Anaplastic large cell lymphoma**			
	**ALK-positive anaplastic large cell lymphoma (ALK+ ALCL)**	*ALK* rearrangements	Appropriate for diagnosis and useful for ALK inhibitors treatment
	**ALK-negative anaplastic large cell lymphoma (ALK– ALCL)**	No* ALK* rearrangements	Appropriate for diagnosis and useful for differential diagnosis of ALK-positive ALCL
*DUSP22-IRF4, TP63* rearrangements		Useful for diagnosis and treatment option (e. g. less intensive in DUSP22 rearrangement ALK-negative ALCL)
**Nodal T-follicular helper (TFH) cell lymphoma**			
	**Nodal TFH cell lymphoma(nTFH), angioimmunoblastic-type, nTFH, follicular type, nTFH, NOS**	*TET2, DNMT3A, IDH2* and* RHOA* mutations	Useful for diagnosis
**Peripheral T-cell lymphomas, NOS**			
	**Peripheral T-cell lymphomas, NOS, PTCL-GATA3 subtype**	Losses 9p, 10p, 17p	Appropriate for diagnosis
	**Peripheral T-cell lymphomas, NOS, PTCL-TBX21 subtype**	*TET2*, *DNMT3A* mutations	Appropriate for diagnosis
**EBV-positive NK/T-cell lymphomas**			
	**EBV-positive nodal T- and NK-cell lymphoma**	*TET2*, *PIK3CD*, *DDX3X* and *STAT3* mutations	Appropriate for diagnosis
	**EBV-positive Extranodal NK/T-cell lymphoma**	Loss 6q21-q25	Appropriate for diagnosis

CN-LOH, copy neutral loss of heterozygosity; inv, inversion; t, translocation; I, isochromosome
